# Survival Analysis in Adult Heart Transplantation: Experience from a Brazilian Single Center

**DOI:** 10.21470/1678-9741-2023-0394

**Published:** 2024-09-03

**Authors:** Diogo Luiz de Magalhães Ferraz, Cristiano Berardo Carneiro da Cunha, Fernando Augusto Marinho dos Santos Figueira, Igor Tiago Correia Silva, Verônica Soares Monteiro, Rodrigo Moreno Dias Carneiro, Bruna Gomes de Castro, Mariana Barreto Requião, Victor de França Oliveira, Patrícia Jaqueline Xavier da Silva, Rodrigo Mezzalira Tchaick, Ana Flávia Paiva Furtado, Maria de Fátima Oliveira da Silva Filha, Renato Correia Fernandes de Souza, Maria Julia Gonçalves de Mello, Rodrigo Melo Gallindo

**Affiliations:** 1 Department of Cardiovascular Surgery, Instituto de Medicina Integral Professor Fernando Figueira, Recife, Pernambuco, Brazil; 2 Cardiovascular Research, Harvard Medical School, Boston, Massachusetts, United States of America; 3 Cardiology, Instituto de Medicina Integral Professor Fernando Figueira, Recife, Pernambuco, Brazil; 4 Organ Transplantation, Instituto de Medicina Integral Professor Fernando Figueira, Recife, Pernambuco, Brazil; 5 Perfusion, Instituto de Medicina Integral Professor Fernando Figueira, Recife, Pernambuco, Brazil; 6 Department of Ecology and Evolutionary Biology, The University of Arizona, Tucson, Arizona, United States of America; 7 Postgraduate Program, Instituto de Medicina Integral Professor Fernando Figueira, Recife, Pernambuco, Brazil

**Keywords:** Survival Rate, Chagas Cardiomyopathy, Overweight, Dilated Cardiomyopathy, Cardiopulmonar Bypass, Caude of Death, Heart Transplantation, Risk Factors

## Abstract

**Introduction:**

Heart transplantation is the gold standard for advanced heart failure treatment. This study examines the survival rates and risk factors for early mortality in adult heart transplant recipients at a Brazilian center.

**Methods:**

This retrospective cohort study involved 255 adult heart transplant patients from a single center in Brazil. Data were collected from medical records and databases including three defined periods (2012-2015, 2016-2019, and 2020-2022). Statistical analysis employed Kaplan-Meier survival curves, Cox proportional hazards analysis for 30-day mortality risk factors, and Log-rank tests.

**Results:**

The recipients were mostly male (74.9%), and the mean age was 46.6 years. Main causes of heart failure were idiopathic dilated cardiomyopathy (33.9%), Chagas cardiomyopathy (18%), and ischemic cardiomyopathy (14.3%). The study revealed an overall survival of 68.1% at one year, 58% at five years, and 40.8% at 10 years after heart transplantation. Survivalimproved significantly over time, combining the most recent periods (2016 to 2022) it was 73.2% in the first year and 63% in five years. The main risk factors for 30-day mortality were longer time on cardiopulmonary bypass, the initial period of transplants (2012 to 2015), older age of the donor, and nutritional status of the donor (overweight or obese). The main causes of death within 30 days post-transplant were infection and primary graft dysfunction.

**Conclusion:**

The survival analysis by period demonstrated that the increased surgical volume, coupled with the team’s experience and modifications to the immunosuppression protocol, contributed to the improved early and mid-term outcomes.

## INTRODUCTION

The progression of cardiovascular disease leads to heart failure (HF), resulting in structural or functional impairment of ventricular filling or blood ejection^[1]^. Advanced chronic HF is defined when traditional treatments are no longer effective. Heart transplantation (HTx) remains the gold standard for the treatment of advanced HF in the absence of contraindications^[2,3]^.

The first human heart transplant was performed in December 1967 in South Africa by Christiaan Barnard at Groote Schuur Hospital^[4]^. There was great enthusiasm at the time; however, due to complications such as rejection and infection, most teams interrupted their transplant programs. In Brazil, after the first three cases carried out by the team led by Drs. Zerbini and Décourt between 1968 and 1969, there was a lapse of 17 years, and from 1984, several centers started their heart transplant programs^[5]^.

According to the registry of the Associação Brasileira de Transplante de Órgãos, a total of 6,108 heart transplants were performed in Brazil until December 2022. Since 2014, the country consistently maintained a surgical volume exceeding 300 heart transplants per year, reaching a peak in 2017 with 380 procedures. However, the coronavirus disease 2019 (COVID-19) pandemic led to a noteworthy decline in transplants in 2020, with only 308 heart transplants performed in Brazil^[6]^.

By analyzing survival curves, the most critical post-transplant periods can be defined in the short, medium, and long terms. Understanding the distribution of causes of death over time can optimize survival, and the identification of risk factors for early death is essential to improve patient care and outcomes in HTx. The objectives of this study are to determine the survival rate of patients undergoing HTx in different periods of the center’s experience and to identify the risk factors for early death.

## METHODS

### Patients

This study is a retrospective cohort; 258 consecutive adult patients who underwent HTx from 2012 to 12/31/2022 at a single center in Brazil were included. Three patients were excluded: one who underwent heart re-transplantation, one due to the etiology of complex congenital heart disease, and one due to a surgical technique other than standard bicaval orthotopic surgery (*situs inversus* patient). The study followed the recommendations of the Strengthening the Reporting of Observational Studies in Epidemiology (or STROBE) guideline^. The research followed the standards of the Declaration of Helsinki, was submitted to the institution’s Research Ethics Committee, and was approved by CAAE number 40888620.4.0000.5201.

The patients underwent bicaval orthotopic heart transplant surgery, using the same techniques throughout the study period. Organ harvesting was carried out after family authorization in donors diagnosed with brain death (BD), through median sternotomy. To collect the heart, the myocardial protection used was the ice-cold crystalloid solution histidine-tryptophan-ketoglutarate at a dose of 20 ml/kg, infused between eight and 10 minutes, into the aortic root after clamping the ascending aorta. After removal, the organs were packed in three sterile plastic bags, placed in a thermal box, and transported to the transplant center, where implant surgeries were performed on recipient patients. The harvesting surgery was performed locally, in hospitals in the same city as the transplant center, or regionally, in cities up to 846 km away, using land and air transport.

Implant surgeries were performed through median sternotomy, using the bicaval orthotopic technique, and cardiopulmonary bypass (CPB).The patients were sent to the immediate postoperative period in the intensive care unit (ICU) and then sent to the ward when in clinical condition until hospital discharge.

### Immunosuppression

Immunosuppression for HTx consisted of triple therapy: corticosteroids, calcineurin inhibitors, and antiproliferatives. The early corticosteroid protocol used until mid-2015 was methylprednisolone (10 mg/kg) intravenously during surgery (5 mg/kg during anesthetic induction and 5 mg/kg after organ reperfusion), and in the first three postoperative days, 10 mg/kg of methylprednisolone, followed by weaning from 100 mg daily to a dose of 100 mg/day. From this dose onwards, the intravenous corticosteroid was changed to oral with prednisone 1 mg/kg/day, gradually weaning until discontinuation after the biopsy in the sixth month post-transplant in patients with a low risk of rejection (non-double transplant, non-sensitized patients and without a history of previous rejection). In highly sensitized patients, induction therapy with thymoglobulin was performed. The other immunosuppressants, cyclosporine (calcineurin inhibitor) and mycophenolate (antiproliferative), were started as soon as the oral route was available, usually on the first postoperative day after extubation.

As of mid-2015, the intravenous corticosteroid protocol was based on the Cleveland Clinic protocol, which consisted of the same initial dose of methylprednisolone (10 mg/kg) during surgery. On the first postoperative day, maintenance was performed with methylprednisolone (125 mg) every eight hours, followed by 20 mg of prednisone on the second day for up to three months, reduced to discontinuation on the sixth month, in patients with a low risk of rejection. During the same period, there was also an update for other oral immunosuppressants, with cyclosporine being replaced by tacrolimus, due to the latter having a faster blood dosage result, allowing better adjustment of immunosuppression.

### Data Collection

Recipient data were obtained from medical records and the database of cardiology, cardiovascular surgery, and heart transplant services. Donor data was obtained through the National Transplant System.

The variables collected were recipient and donor age, sex, weight, and height; recipient comorbidities (diabetes and hypertension), panel reactive antibody, priority status, use of mechanical circulatory support before transplantation, graft ischemic time, city of retrieval operation; date of transplant, final date (death or censored), death, cause of death; donor history of cardiorespiratory resuscitation, use of vasoactive drugs, use of antibiotics, serum sodium, and hospital length of stay. The calculated variables were recipient and donor body mass index.

Three periods of transplantation were defined: period 1 (2012 to 2015), related to the initial experience; period 2 (2016 to 2019), after changing the early corticosteroid protocol; and period 3 (2020 to 2022), which occurred during the COVID-19 pandemic. Death within 30 days after transplantation was defined as early mortality. Death occurring after 30 days of HTx was considered as late mortality.

### Statistical Analysis

Survival time was calculated from the date of transplantation to the date of death or until censoring, considering the date of the last consultation for patients lost to follow-up. Missing data were excluded depending on the variable under analysis.

To identify risk factors for 30-day mortality, the population was categorized into two groups, survivors and non-survivors at 30 days, and the analysis was conducted using Cox proportional hazards modeling.

The Kaplan-Meier method was used to obtain survival curves. Based on these analyses, comparisons were made between groups: HTx periods, donors and recipients of the opposite sex, transplantation with ABO-heterogeneous group compatible, and age groups (< 60 and ≥ 60 years old). The differences were assessed using the Log-rank test. A value of *P*<0.05 was considered statistically significant. All statistical analyses were performed using Stata software, version 18.0 (Stata Corp).

## RESULTS

### Study Population

In this cohort, data from 255 adult patients who underwent HTx between 2012 and 2022 were analyzed. The mean age of the recipients was 46.6 years, with 74.9% being male. The clinical characteristics of the studied population are shown in Table 1. Most patients undergoing HTx had blood group O (48.2%), followed by groups A (38.0%), B (8.2%), and AB (5.5%). Donors had the following proportions: O (63.1%), A (31.4%), B (5.1%), and AB (0.4%). There was a non-identical ABO (only compatible) group-matched transplant rate of 20% (51 patients).

**Table 1:** Characteristics of heart transplant recipients and comparison between survivors and non-survivors in 30-day mortality.

Recipient’s variables	All cohort (N = 255)	HTx survivors (N = 221)	HTx non-survivors (N = 34)	HR	95% CI	*P*-value
Age (years)				1.02	1.00-1.05	0.090
Mean ± SD	46.6 ± 12.9	46.1 ± 13.0	50.3 ± 11.5			
Median (IQR)	49 (39-56)	48 (37-56)	51 (44-59)			
Age group ≥ 60 years	45 (17.6%)	37 (16.7%)	8 (23.5%)	1.44	0.65-3.18	0.366
Sex						
Male	191 (74.9%)	168 (76.0%)	23 (67.6%)	1.00		
Female	64 (25.1%)	53 (24.0%)	11 (32.4%)	1.47	0.72-3.01	0.294
BMI (kg/m^2^)				1.03	0.95-1.11	0.437
Mean ± SD	23.5 ± 4.0	23.4 ± 4.1	24.1 ± 4.1			
Median (IQR)	23.0 (20.8-25.9)	22.9 (20.8-25.8)	24.1 (21.0-27.1)			
Nutritional status						
BMI (kg/m^2^)						
BMI < 18.5	23 (9.0%)	20 (9.1%)	3 (8.8%)	1.22	0.36-4.18	0.753
BMI = 18.5-24.9	146 (57.3%)	130 (58.8%)	16 (47.1%)	1.00		
BMI ≥ 25	86 (86.7%)	71 (32.1%)	15 (44.1%)	1.61	0.80-3.26	0.184
Diabetes (N=243)	37 (15.2%)	33 (15.1%)	4 (16.0%)	1.05	0.36-3.07	0.923
Hypertension (N=243)	83 (34.2%)	77 (35.3%)	6 (24.0%)	0.60	0.23-1.50	0.277
PRA > 0% (N=216)	33 (15.3%)	27 (14.0%)	6 (26.1%)	2.09	0.82-5.31	0.120
Priority status	105 (41.2%)	87 (39.4%)	18 (52.9%)	1.70	0.87-3.33	0.123
MCS	12 (4.7%)	9 (4.1%)	3 (8.8%)	2.24	0.68-7.33	0.183
Ischemic time (min.) (N=192)				1.00	0.99-1.00	0.392
Mean ± SD	177.0 ± 70.1	175.6 ± 69.8	190.1 ± 73.6			
Median (IQR)	187.5 (110-240)	185 (108-240)	235 (20-250)			
Prolonged ischemic time > 240 min. (N=192)	45 (23.4%)	39 (22.5%)	6 (31.6%)	1.54	0.59-4.07	0.377
Retrieval operation						
Local	131 (51.4%)	115 (52.0%)	16 (47.1%)	1.00		
Regional	124 (48.6%)	106 (48.0%)	18 (52.9%)	1.21	0.62-2.37	0.581
CPB time (min.) (N=186)				1.01	1.00-1.02	0.001
Mean ± SD	126.5 ± 41.4	123.2 ± 34.5	156.7 ± 74.9			
Median (IQR)	117 (100-138)	116 (100-135)	120 (105-193)			
HTx period						
2012-2015	75 (29.4%)	58 (26.3%)	17 (50.0%)	1.00		
2016-2019	124 (48.6%)	111 (50.2%)	13 (38.2%)	0.43	0.21-0.88	0.021
2020-2022	56 (22.0%)	52 (23.5%)	4 (11.8%)	0.29	0.10-0.85	0.024

BMI=body mass index; CI=confidence interval; CPB=cardiopulmonary bypass; HR=hazard ratio; HTx=heart transplantation; IQR=inter-quartile range; MCS=mechanical circulatory support; PRA=panel reactive antibody; SD=standard deviation

**Fig. 1:**
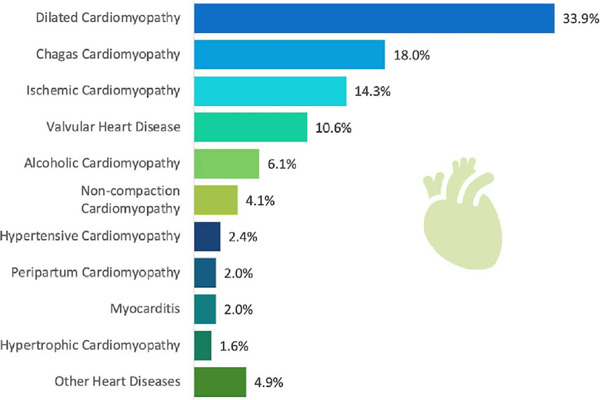
Heart failure etiology among heart transplant recipients from 2012 to 2022.

The etiologies of HF that led to the indication for HTx are listed in Figure 1. Idiopathic dilated cardiomyopathy was the main cause of indication for HTx, followed by chagasic and ischemic cardiomyopathy. Causes classified as “other” in Figure 1 included storage diseases such as amyloidosis or hemochromatosis, arrhythmogenic right ventricular dysplasia, leptospirosis, tachycardiomyopathy, and hyperthyroidism. No data were found in the medical records regarding the etiology of HF in 10 patients, who are not included in this analysis.

**Fig. 2:**
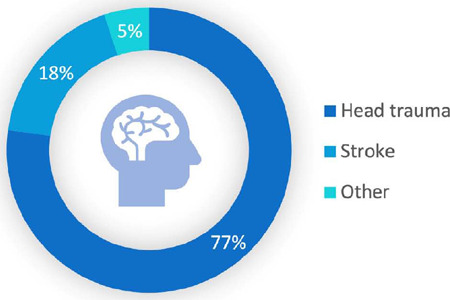
Causes of brain death in heart donors between 2012 and2022.

The general characteristics of heart donors are described in Table 2. The main cause of BD in donors was traumatic brain injury (TBI), followed by stroke as seen in Figure 2. Data classified as “other” include cerebral hypoxia, intracranial hypertension, exogenous intoxication, meningoencephalitis, brain abscess, and brain tumor.

**Table 2:** Characteristics of heart transplant donors and comparison between groups of surviving and non-surviving recipients in 30-day mortality.

Donor variables	All cohort (N =255)	HTx survivors (N = 221)	HTx non-survivors (N = 34)	HR	95% CI	*P*-value
Age (years)				1.06	1.03-1.10	0.001
Mean ± SD	29.6 ± 9.8	28.8 ± 9.5	35.1 ± 9.8			
Median (IQR)	28 (21-38)	27 (21-37)	37 (28-44)			
Sex						
Male	217 (85.1%)	188 (85.1%)	29 (85.3%)	1.00		
Female	38 (14.9%)	33 (14.9%)	5 (14.7%)	0.99	0.39-2.57	0.993
BMI (kg/m^2^) (N = 254)				1.07	0.98-1.17	0.145
Mean ± SD	25.7 ± 3.3	25.6 ± 3.4	26.5 ± 2.6			
Median (IQR)	25.3 (23.6-27.7)	25.1 (23.4-27.7)	26.2 (24.7-28.3)			
Nutritional status						
BMI (kg/m^2^) (N = 254)						
BMI < 18.5	0 (0%)	0 (0%)	0 (0%)			
BMI = 18.5-24.9	119 (46.9%)	109 (49.5%)	10 (29.4%)	1.00		
BMI ≥ 25	135 (53.1%)	111 (50.5%)	24 (70.6%)	2.19	1.05-4.58	0.037
History of CPR (N=254)	36 (14.5%)	31 (14.1%)	5 (14.7%)	1.04	0.41-2.71	0.923
Use of vasoactive drugs (N=254)	222 (87.4%)	193 (87.7%)	29 (85.3%)	0.83	0.32-2.1	0.699
Use of antibiotics (N=254)	153 (60.2%)	135 (61.4%)	18 (52.9%)	0.71	0.36-1.40	0.328
Na > 164 mEq/L (N=254)	69 (27.2%)	62 (28.2%)	7 (20.6%)	0.67	0.29-1.54	0.347
Na (mEq/L) (N=254)				0.99	0.97-1.01	0.386
Mean ± SD	157.2 ± 13.9	157.5 ± 13.9	155.4 ± 14.0			
Median (IQR)	157 (147-166)	158 (147-166.5)	154.5 (147-163)			
Hospital length of stay (days) (N=254)				1.06	1.00-1.13	0.053
Mean ± SD	4.8 ± 4.2	4.6 ± 3.2	5.8 ± 8.1			
Median (IQR)	4 (3-6)	4 (3-6)	3.5 (3-6)			

BMI=body mass index; CI=confidence interval; CPR=cardiopulmonary resuscitation; HR=hazard ratio; HTx=heart transplantation; IQR=interquartile range; Na=serum sodium; SD=standard deviation

### Early Results

In total, 34 patients (13.3%) died within 30 days postoperatively. There was a significant reduction in 30-day lethality in the analysis by periods: period 1 (2012 to 2015) 22.7%, period 2 (2016 to 2019) 10.4%, and period 3 (2020 to 2022) 7.14%, with *P*=0.011. When compared to period 1, a lower risk of early death was founded for both period 2 (hazard ratio [HR] 0.43; 95% confidence interval [CI] 0.19-0.95) and period 3 (HR 0.29; 95% CI 0.49-0.74).

**Fig. 3:**
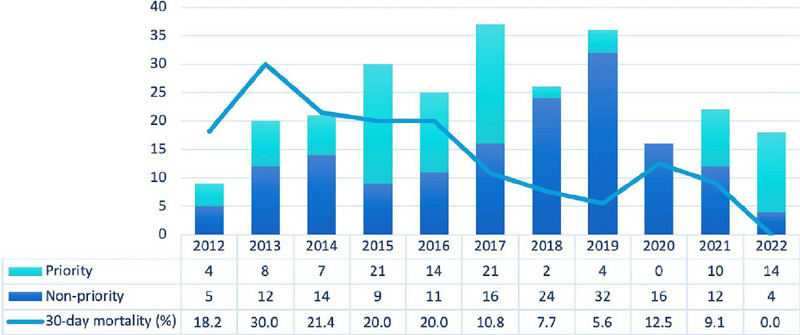
Distribution of heart transplants performed between 2012 and 2022, showing the prioritization status and the 30-day mortality curve per year.

The distribution of heart transplants performed over the years and their relationship with 30-day mortality, which highlights the service’s learning curve, is shown in Figure 3. Regarding the factors that increase the risk of mortality in 30 days, longer time on CPB, the initial period (2012-2015) of transplants, older age of the donor, and nutritional status of the donor (overweight or obese) were founded in the univariable analysis, as shown in Tables 1 2.

In multivariable analysis using Cox regression, the first model included all variables with a *P*-value < 0.300. In this analysis, it was found that CPB time (HR 1.01; 95% CI 1.002-1.014; *P*=0.011) and donor age (HR 1.08; 95% CI 1.023-1.139; *P*=0.005) presented statistical significance. However, as CPB time had 27% missing data, only 186 patients were included in this analysis. The second multivariable analysis model included variables with a *P*-value < 0.300, excluding CPB time. At the end of this analysis, 255 study patients were included, and statistical significance was reached for the donor’s age and for the most recent transplant periods, as shown in Table 3.

**Table 3:** Multivariable analysis of risk factors for mortality within 30 days after heart transplantation.

Variables	All cohort (N = 255)	HTx survivors (N = 221)	HTx non-survivors (N = 34)	HR	95% CI	*P*-value
Donor age (years)				1.06	1.02-1.10	0.001
Mean ± SD	29.6 ± 9.8	28.8 ± 9.5	35.1 ± 9.8			
Median (IQR)	28 (21-38)	27 (21-37)	37 (28-44)			
HTx period						
2012-2015	75 (29.4%)	58 (26.3%)	17 (50.0%)	1.00		
2016-2019	124 (48.6%)	111 (50.2%)	13 (38.2%)	0.41	0.20-0.85	0.016
2020-2022	56 (22.0%)	52 (23.5%)	4 (11.8%)	0.32	0.11-0.96	0.042

CI=confidence interval; HR=hazard ratio; HTx=heart transplantation; IQR=interquartile range; SD=standard deviation

### Survival Analysis

**Fig. 4:**
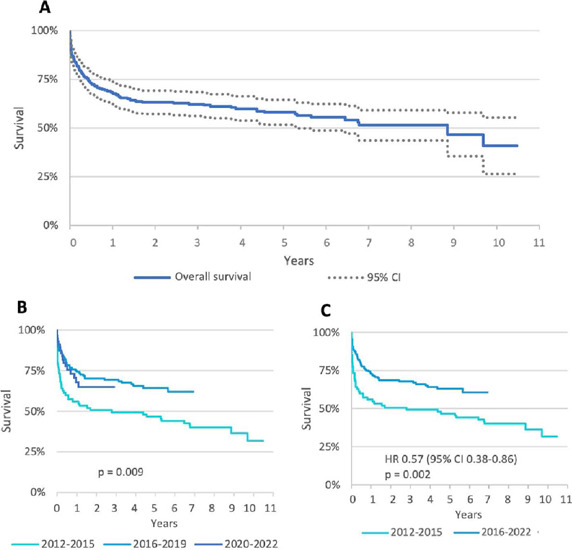
Kaplan-Meier survival curves of heart transplant recipients between 2012 and 2022. A) Overall survival; B) survival curves comparing the three study periods; C) survival curves comparing two periods (union of the most recent periods). CI=confidence interval; HR=hazard ratio.

The mean and median follow-up times were 3.1 and 2.4 years, respectively. The longest follow-up time was 10.5 years, and 108 deaths occurred during this period. Overall survival for one, five, and 10 years was 68.1%, 58.0%, and 40.8%, respectively. The median survival time was 8.8 years (Figure 4A).

Analyzing survival by transplant periods, a difference was found between periods 1 and 2 with statistical significance (*P*=0.009). When compared to period 1, we found HR 0.55 and 95% CI 0.360.85 for period 2 and HR 0.64 and 95% CI 0.36-1.12 for period 3 (Figure 4B). Survival in the most recent periods (from 2016 to 2022) was 73.2% in the first year and 63% in five years (Figure 4C).

In other subgroup analyses, there was no difference in survival in transplants with donors and recipients of the opposite sex, as well as patients who received transplant ABO-heterogeneous group compatible. However, there was a difference in the analysis by age group, with patients aged 60 years or older having a median survival of 1.14 year, while younger patients had a median of 8.8 years (P=0.0045).

### Causes of Death

In this cohort of 255 individuals, 108 deaths occurred in 10.5 years of follow-up. The main cause of death was infection (including bloodstream, lung, sepsis, etc.) in 47 patients. The second most frequent cause was COVID-19, with 15 patients, and the third cause was primary graft dysfunction, with seven patients. The following were classified as “other”: stroke, sudden death, neoplasia, hemorrhagic shock, diabetic ketoacidosis, recurrence of Chagas disease, aneurysm rupture, or undetermined cause.

**Fig. 5:**
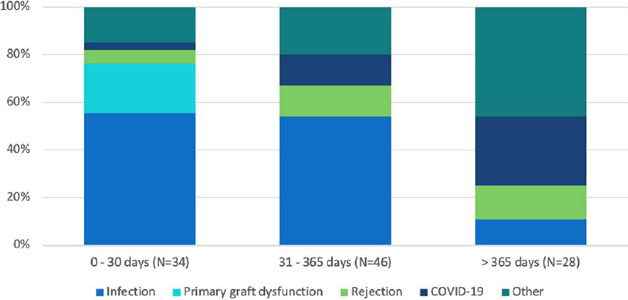
Causes of death in adult heart transplant recipients between 2012 and 2022. COVID-19=coronavirus disease 2019.

In the first 30 postoperative days, 56% of deaths occurred due to infectious causes, 21% due to primary graft dysfunction, 6% due to rejection, and 3% due to COVID-19. Between 31 days and one year after transplant, 54% died from infection, 13% from rejection, and 13% from COVID-19. Patients older than one year died from infection in 11%, from rejection in 14%, from COVID-19 in 29%, and from other causes in 46% (Figure 5).

## DISCUSSION

Post-heart transplant survival analysis involves a series of factors: the experience of the transplant center, the profile of recipient patients with their different levels of severity, the profile of organ donors, postoperative management in the ICU, adjustments of immunosuppressants, postoperative complications of infection, rejection, vascular graft disease, and neoplasms.

When comparing the recipient’s basic characteristics with other published Brazilian studies, Chagas disease was the second most common etiology of heart transplant recipients in this study, while in other centers, after idiopathic dilated heart disease, the second most common etiology is ischemic.

In a study of a center in São Paulo, the etiologies of HF were dilated cardiomyopathy (45.6%), ischemic (25%), and chagasic (22.8%) in a series of patients until 1998^[5]^. A study in the city of Fortaleza presented the following causes of cardiomyopathy: idiopathic (32.2%), ischemic (25.5%), and chagasic (17.5%)^[8]^. While in this study carried out in Recife, the main etiologies were idiopathic (33.9%), chagasic (18%), and ischemic (14%).

In an epidemiological study published in 2011, the state of Pernambuco ranked second in Brazil in acute cases of Chagas disease with 274 cases (2001 to 2006), behind Bahia with 441 cases^[9]^. A study of the prevalence of Chagas disease in Brazil, published in 2014, presented Bahia with 2.4% and Pernambuco with 9.1%^[10]^. In addition to the high prevalence of Chagas disease in Pernambuco, the hospital also receives patients from Bahia for HTx, justifying this emphasis on chagasic heart disease among heart recipients in this population.

Regarding the characteristics of heart donors, we see a difference when comparing with international data. According to the International Society for Heart and Lung Transplantation (or ISHLT) registry, with more than 90% of the data coming from transplants performed in the United States of America and Europe, the mean age of donors is 35 years old, and the causes of BD were TBI (45%), stroke (24%), and others (30%)^[11]^. In our study, the mean age was 29 years old, and the causes were TBI (77%), stroke (18%), and others (5%).

This higher rate ofTBI in Brazil reflects the consequences of reckless driving, with high rates of automobile accidents and in addition to those caused by firearms, bladed weapons, and blunt trauma due to urban violence.

The main cause of early mortality in this study in post-heart transplant patients was infections, which caused 56% of deaths in the first 30 days. We can compare this data with a European study, which showed 10% of deaths due to infection within 30 days^[12]^. Given immunosuppression and the need for hospitalization while on the waiting list, heart transplant recipients are at high risk of contracting hospital-acquired infections. Bloodstream infections, related to catheters and circulatory assistance devices, urinary tract infections, and pneumonia associated with mechanical ventilation can progress to sepsis in the context of post-transplant immunosuppression and lead to death.

Deaths due to acute rejection in the present study showed a lower percentage, 6% within 30 days and 13% from 31 days to one year, when compared to the same European study with 28% and 32%, respectively^[12]^.

Primary graft dysfunction was the second cause of early death in this study (21%). It is a multifactorial condition with its pathophysiology not yet well understood, but it presents some known risk factors such as recipient patients using vasoactive drugs or mechanical circulatory assistance, elderly donors, and prolonged ischemia time.

In the univariable analysis, one of the factors that presented statistical significance was the CPB time. However, there is a bias in this variable, considering that a longer CPB time depends on several factors such as: the difficulty in cardiectomy of the recipient (mainly in cases of previous median sternotomy), the heart implantation technique following the anastomoses, as well as the reperfusion time of the organ necessary to restore the biventricular cardiac function of the graft. It is necessary to maintain the patient on CPB until adequate hemodynamic stability is achieved, with an adjustment of vasoactive drugs and an assessment of the need for circulatory assistance in case of primary graft dysfunction.

Therefore, the prolonged CPB time reflects a technically more difficult procedure, and the patient is also subject to the consequences of the CPB itself, with a greater risk of presenting systemic inflammatory response syndrome, platelet dysfunction, and hemolysis.

Patients who received hearts from donors with a BMI ≥ 25 kg/m^2^ and who were older had worse 30-day survival results in the univariable analysis. Overweight may be related to other comorbidities not listed, such as hypertension and diabetes mellitus in the donor, which may favor more primary graft dysfunction.

Analysis between study periods demonstrated that initial 30-day mortality was higher, and a progressive and significant reduction in subsequent periods, reaching a rate < 10% as of 2018. The success of the learning curve is due to constant updating of the team, together with training and gaining experience from professionals in different sectors of the hospital, which improves the clinical evaluation of both donor and recipient and the exchange of experiences between transplant centers in Brazil.

The institution that developed this study has become a high-volume heart transplant center, performing more than 20 transplants per year, and this has resulted in a significant improvement in outcomes. An important point to be highlighted was the change in the immunosuppression protocol carried out in mid-2015, with lower doses of corticosteroids, which drastically reduced complications of infection and early mortality, also reflecting survival in the mid-term.

The third period of the study (2020 to 2022) was marked by the COVID-19 pandemic, with airway disease caused by the severe acute respiratory syndrome coronavirus 2 (SARS-CoV-2) virus. Due to the large number of infected people, there was a significant drop in the number of heart transplants performed.

In addition to there being restrictions on the care of patients with HF in hospitals with the closure of outpatient care and emergency rooms full of patients with severe acute respiratory syndrome, the low circulation of people in cities also led to a significant drop in organ donations.

In 2020, transplants were only performed in patients non-prioritized in this cohort, which highlighted the difficulty in accessing the evaluating and listing of more critical patients. The longer time to obtain donors also led to a greater possibility of death on the waiting list. Associated with this, there was a change in the downward trend in the 30-day mortality rate that year.

In 2021, with the start of vaccination against SARS-CoV-2 in Brazil and the institution of specific protocols for testing donors and recipients, there was a drop in the number of COVID-19 cases and an increase in the number of heart transplants performed in our service, due to increasing safety when carrying out the procedure. In 2022, with the pandemic still ongoing but stable, there was the arrival of rapid SARS-CoV-2 antigen tests and the advancement of vaccination. This improves the access of patients with HF to the hospital. Considering the worsening of these patients’ conditions due to a lack of follow-up, the vast majority (77.8%) of transplants performed at the institution in 2022 were performed on priority recipients. However, the mark of 0% mortality in 30 days was reached this year.

The analysis of medium-term survival published with Brazilian data shows similarities between regions. In a study published in 2021 with 2,197 patients from Brazil, survival was 70.9% in one year, 59.5% in five years, and 45.1% in 10 years, with a median survival time of 8.3 years^[13]^.

Two studies in hospitals in São Paulo showed survival rates at one and five years of 70.4% and 59.9% at the Instituto Dante Pazzanese de Cardiologia^[14]^ and 71% and 54.4% with the team from the Universidade Federal de São Paulo. A published study from Hospital de Messejana in Ceará showed overall survival rates of 73% and 60% at one and five years, respectively. Our study revealed overall survival rates at one and five years of 68% and 58%. These data were negatively impacted by the initial results of the learning curve but were also strongly affected by the pandemic. COVID-19 was responsible for early postoperative mortality, accounting for 13% of deaths between 30 days and one year, but primarily for late postoperative mortality, being the cause in 29% of deaths in patients beyond one year after transplant.

Patients in the age group of 60 years or older did not have a difference in 30-day mortality. However, the result of the overall survival curve was significantly worse compared to younger individuals. One of the factors that may contribute to this group of patients is frailty. In a study conducted in Australia, pre-transplant frailty status was an independent risk factor for increased mortality and length of stay after cardiac transplantation^[15]^.

### Limitations

The limitations of the study are related to its retrospective nature, being from a single center, and having a relatively small sample. We will continue collecting data for an analysis with more participants and longer follow-up.

## CONCLUSION

Adult HTx has shown a significant decrease in early mortality over the years. The third period of the study (2020 to 2022) was marked by the COVID-19 pandemic, which adversely affected the annual transplant numbers. The survival analysis by period demonstrated that the increased surgical volume, coupled with the team’s experience and modifications to the immunosuppression protocol, contributed to the improved early and mid-term outcomes.

## Abbreviations, Acronyms & Symbols

BD: Brain death
BMI: Body mass index
CI: Confidence interval
COVID-19: Coronavirus disease 2019
CPB: Cardiopulmonary bypass
CPR: Cardiopulmonary resuscitation
HF: Heart failure
HR: Hazard ratio
HTx: Heart transplantation
ICU: Intensive care unit
IQR: Interquartile range
MCS: Mechanical circulatory support
Na: Serum sodium
PRA: Panel reactive antibody
SARS-CoV-2: Severe acute respiratory syndrome coronavirus 2
SD: Standard deviation
TBI: Traumatic brain injury
